# Exploring Inflammatory Asthma Phenotypes: Proteomic Signatures in Serum and Induced Sputum

**DOI:** 10.3390/ijms25063501

**Published:** 2024-03-20

**Authors:** Larissa Prado Maia, Thulio Marquez Cunha, Paula Souza Santos, Mario Machado Martins, Peter Briza, Fatima Ferreira, Maria Marta Amorim, Lilian Ballini Caetano, Camyla Fernandes Farias, Ilka Lopes Santoro, Ana Luisa Godoy Fernandes, Luiz Ricardo Goulart

**Affiliations:** 1 Laboratory of Nanobiotechnology, Institute of Biotechnology, Federal University of Uberlandia, Uberlandia 38405-320, Brazil; paulasantos.bio@gmail.com (P.S.S.); mariomm1988@yahoo.com.br (M.M.M.); 2 Department of Pulmonology, School of Medicine, Federal University of Uberlandia, Uberlandia 38405-320, Brazil; thcunha@yahoo.com.br; 3 Department of Biosciences and Medical Biology, University of Salzburg, 5020 Salzburg, Austria; peter.briza@plus.ac.at (P.B.);; 4 Respiratory Division, Escola Paulista de Medicina, Universidade Federal de São Paulo, Sao Paulo 04023-062, Brazil; martafamorim@gmail.com (M.M.A.); elilian@terra.com.br (L.B.C.); camyla.ff@gmail.com (C.F.F.); ilkasantoro@gmail.com (I.L.S.); analuisa.pneumo@gmail.com (A.L.G.F.)

**Keywords:** asthma phenotyping, inflammatory profile, proteomic analysis

## Abstract

Asthma drug responses may differ due to inflammatory mechanisms triggered by the immune cells in the pulmonary microenvironment. Thus, asthma phenotyping based on the local inflammatory profile may aid in treatment definition and the identification of new therapeutic targets. Here, we investigated protein profiles of induced sputum and serum from asthma patients classified into eosinophilic, neutrophilic, mixed granulocytic, and paucigranulocytic asthma, according to inflammatory phenotypes. Proteomic analyses were performed using an ultra-performance liquid chromatography (ultra-HPLC) system coupled to the Q Exactive Hybrid Quadrupole Orbitrap Mass Spectrometer. Fifty-two (52) proteins showed significant differences in induced sputum among the groups, while only 12 were altered in patients’ sera. Five proteins in the induced sputum were able to discriminate all phenotypic groups, while four proteins in the serum could differentiate all except the neutrophilic from the paucigranulocytic inflammatory pattern. This is the first report on comparative proteomics of inflammatory asthma phenotypes in both sputum and serum samples. We have identified a potential five-biomarker panel that may be able to discriminate all four inflammatory phenotypes in sputum. These findings not only provide insights into potential therapeutic targets but also emphasize the potential for personalized treatment approaches in asthma management.

## 1. Introduction

Asthma is a chronic inflammatory disorder of the airways, characterized by respiratory symptoms, bronchial hyperresponsiveness, and variable airflow limitation [1]. Although asthma phenotypes are not well established due to the heterogeneity of asthma, it is possible to classify patients by clinical, physiological, and pathological characteristics. Characterizing these patients based on the predominant inflammatory cell type in the airways is an important phenotyping practice. These classifications can improve treatment, since patients with different phenotypes frequently have different drug responses [2,3].

Eosinophilic asthma is characterized by a high number of eosinophils in sputum, more commonly present in allergic inflammation. This finding is associated with production of immunoglobulin (Ig) E and cytokines related to T-helper cell type 2 (Th2) immune response [4,5,6]. Neutrophilic asthma, characterized by high levels of neutrophils in sputum, seems to be associated with resistance to treatment with corticosteroids and a higher severity of the disease. It is also linked with Th1- and Th17-associated inflammation [7,8,9]. Mixed granulocytic asthma consists of concomitant eosinophilic and neutrophilic inflammation [5]. These patients usually have lower lung function and severe airway hyperresponsiveness. On the other hand, paucigranulocytic asthma consists of a low inflammatory cell count in sputum patients. Commonly, these patients are non-allergic and do not have severely affected pulmonary function [10]. Discussions about inflammatory phenotypes have enhanced in recent years. However, some data are inconsistent because of a lack of information, especially for non-eosinophilic asthma [11,12,13].

Although many biomarkers with clinical applicability have been proposed to phenotype asthma related to the Th2-mediated pathway, only a few have shown great potential for the diagnosis and prognosis of this phenotype. The fraction of exhaled nitric oxide (FENO) and blood eosinophil count have been used in the diagnosis of Th2-high asthma. However, setting a unique threshold for blood eosinophil count applicable for all patients is not yet possible [14,15,16]. Furthermore, periostin and total and specific IgE levels were also described with this aim [17]; however, their use remains controversial [18]. Moreover, there are only a few potentially useful biomarkers for non-Th2 asthma subtypes, with no drug target success [19,20]. Therefore, the search for personalized and effective therapy for asthma management targeting the pathophysiology of patients has critically influenced the research on new biomarkers to better phenotype the disease [2,15]. 

Alterations in protein expression can improve the understanding of asthma inflammatory patterns and the development of biomarkers suitable for clinical assays for phenotyping. Knowing this, we applied label-free quantitative proteomics to determine global proteins of induced sputum and serum samples from patients with eosinophilic asthma, neutrophilic asthma, mixed granulocytic asthma, and paucigranulocytic asthma. Therefore, we sought to investigate differentially expressed proteins among these groups and identify shared proteins present in both induced sputum and serum. These identified proteins could eventually be integrated into point-of-care biosensor platforms for asthma phenotyping, ultimately enhancing treatment strategies. Additionally, they may be explored as potential targets for therapy. 

## 2. Results

### 2.1. Clinical Characteristics

The clinical and demographic characteristics of the subjects are reported in Table 1. There was no significant difference in age, gender, body mass index (BMI), age at onset, skin prick test positivity, and forced expiratory volume in 1 s (FEV1) between asthma phenotypes. The sputum eosinophils and neutrophils count used to classify the patients showed a significant difference (*p* < 0.0001). It is worth noting that the cut-off point used was 3% or greater for eosinophils and 65% or greater for neutrophils. As expected, the eosinophil count showed differences when comparing the eosinophilic group with the neutrophilic and paucigranulocytic groups, since individuals in the latter two groups did not have eosinophilia. Similarly, when analyzing the neutrophil count in sputum, the neutrophilic group showed a significant difference compared to the eosinophilic and paucigranulocytic groups. Surprisingly, there was also no significant difference in blood eosinophil and neutrophil counts between the analyzed groups. Asthma severity was classified based on the strategy coded by the GINA guidelines. None of the patients were in GINA steps 1 and 2, one patient in the paucigranulocytic group was in GINA step 3, and all other patients were in GINA steps 4 and 5. All patients were under treatment with inhaled corticosteroids at the time of sample collection, and there were no significant differences in the doses used between the analyzed groups.

### 2.2. Proteomic Profiling

The protein profile of the patients’ serum and induced sputum was obtained using the proteomic technique via mass spectrometry. A total of 2266 proteins were identified in sputum samples, while a fewer number, 1373 proteins, was found in serum samples. Most of the proteins were detected in all groups but in less than half of the patients of each group. Only the eosinophilic group presented an exclusive protein, the matrix metalloproteinase 21 (MMP21) found in the serum samples, which was not present in the other groups. 

**Figure 1:**
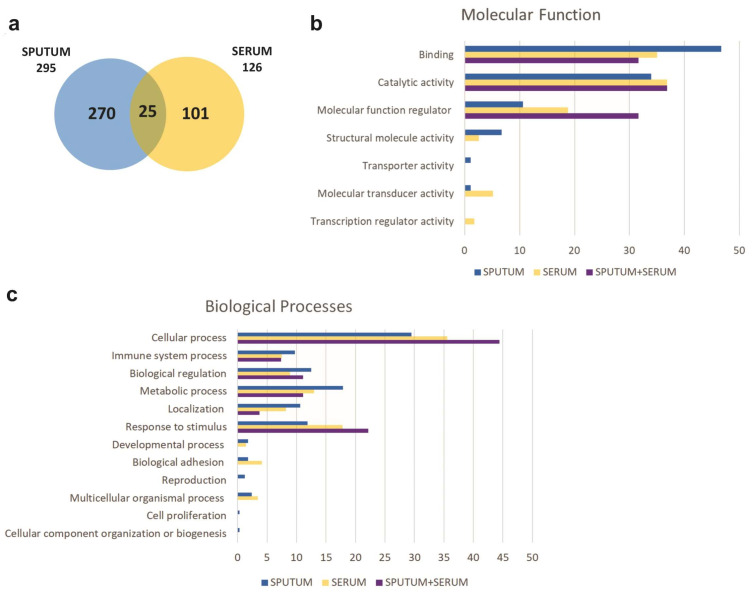
Proteins identified in the sputum and serum of patients with asthma. (**a**) Venn diagram displaying proteins identified in the induced sputum and serum of patients with asthma. (**b**) Molecular function of proteins identified in induced sputum, serum, and in both biological samples via PANTHER gene ontology search, shown as percentage. (**c**) Biological processes of proteins identified in induced sputum, serum, and in both biological samples via PANTHER gene ontology search, shown as percentage.

Differentially expressed serum and sputum proteins among the study groups were investigated only considering the proteins that were present in all groups, in at least half of the individuals in each group. Therefore, 295 proteins were analyzed in sputum samples and 126 proteins in serum samples, 25 of which were common to both types of samples, as illustrated by the Venn Diagrams (Figure 1a). The gene ontology database search showed the molecular function (Figure 1b) and the biological processes (Figure 1c) performed by the identified proteins. The main molecular functions of proteins are binding (46.7% in sputum and 35.0% in serum), catalytic activity (33.9% in sputum and 36.8% in serum), and molecular function regulator (10.6% in sputum and 18.8% in serum). These proteins play a role in several biological processes, the most frequent of which are cellular processes (29.5% in sputum and 35.6% in serum), metabolic processes (17.9% in sputum and 13.0% in serum), biological regulation (12.5% in sputum and 8.9% in serum), response to stimulus (11.9% in sputum and 17.8% in serum), localization (10.6% in sputum and 8.2 in serum), and immune system processes (9.7% in sputum and 7.5% in serum).

### 2.3. Identification of Biomarkers

**Figure 2:**
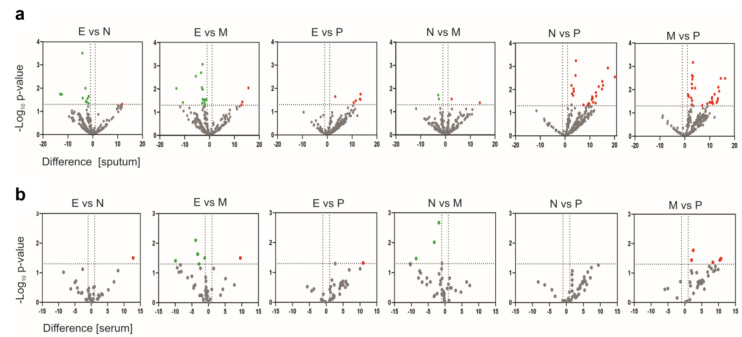
Volcano plot showing the difference and significance (−log10 *p*-value) of the differentially expressed proteins in sputum (**a**) and serum (**b**) for the six group comparisons. Significantly upregulated proteins are marked in red, significantly downregulated proteins are marked in green and the not significant are marked in gray. E, eosinophilic asthma; N, neutrophilic asthma; P, paucigranulocytic asthma; M, mixed granulocytic asthma.

We identified 52 differentially expressed proteins in sputum (Supplementary Table S1), among which the majority were upregulated in neutrophilic and mixed phenotypes, and 12 proteins were different in serum (Supplementary Table S2). Of these, only one protein (Cysteine-rich secretory protein 3-CRISP3) overlapped in serum and induced sputum with a different pattern of change. Volcano plots of difference and significance showed clear differences between the paucigranulocytic group and the neutrophilic and mixed granulocytic groups for the induced sputum data (Figure 2a). It was also discovered that the proteome of eosinophilic patients had dysregulated proteins concerning neutrophilic and mixed, but it was not significantly different from paucigranulocytic. The neutrophilic and mixed granulocytic groups showed little statistically significant difference in protein expression. However, such a clear difference was not observed in serum samples (Figure 2b). The highest difference occurred between eosinophilic and paucigranulocytic regarding the mixed granulocytic group. No differentially abundant protein between the proteome of neutrophilic and paucigranulocytic patients was identified. 

**Figure 3:**
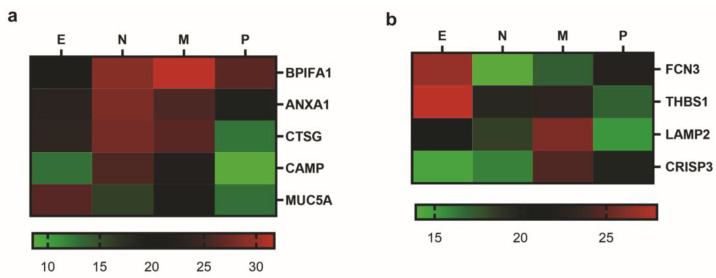
Heat map showing normalized intensity of differentially expressed proteins. Induced sputum (**a**) and serum (**b**) of eosinophilic (E), neutrophilic (N), mixed granulocytic (M), and paucigranulocytic (P) asthma.

Among the significantly dysregulated proteins, a panel of five proteins identified in induced sputum samples differentiates the four phenotype groups (Figure 3a). BPI fold-containing family A member 1 (BPIFA1) was strongly upregulated in the mixed granulocytic group compared with the eosinophilic and neutrophilic groups, whereas mucin 5AC (MUC5AC) demonstrated a significant increase in the eosinophilic group compared with the paucigranulocytic group. Cathelicidin antimicrobial peptide (CAMP) was upregulated in the neutrophilic group, different from the eosinophilic and paucigranulocytic groups, and cathepsin G (CTSG) was also decreased in the paucigranulocytic group compared with the neutrophilic and mixed granulocytic groups. Furthermore, a slight difference in annexin A1 (ANXA1) expression enabled us to distinguish between the neutrophilic and mixed granulocytic groups. 

Another four proteins selected from serum samples were able to discriminate almost all groups, except the paucigranulocytic and neutrophilic groups (Figure 3b). Ficolin-3 (FCN3) and thrombospondin-1 (THBS1) were upregulated in the eosinophilic group in such a way that expression levels of FCN3 could distinguish the eosinophilic from the neutrophilic and mixed granulocytic groups, while THBS1 could distinguish the eosinophilic from the paucigranulocytic group. By contrast, lysosome-associated membrane glycoprotein 2 (LAMP2) and cysteine-rich secretory protein 3 (CRISP3) were significantly increased in the mixed granulocytic group, compared with the paucigranulocytic group and the eosinophilic and neutrophilic groups, respectively.

### 2.4. Protein Networks

**Figure 4:**
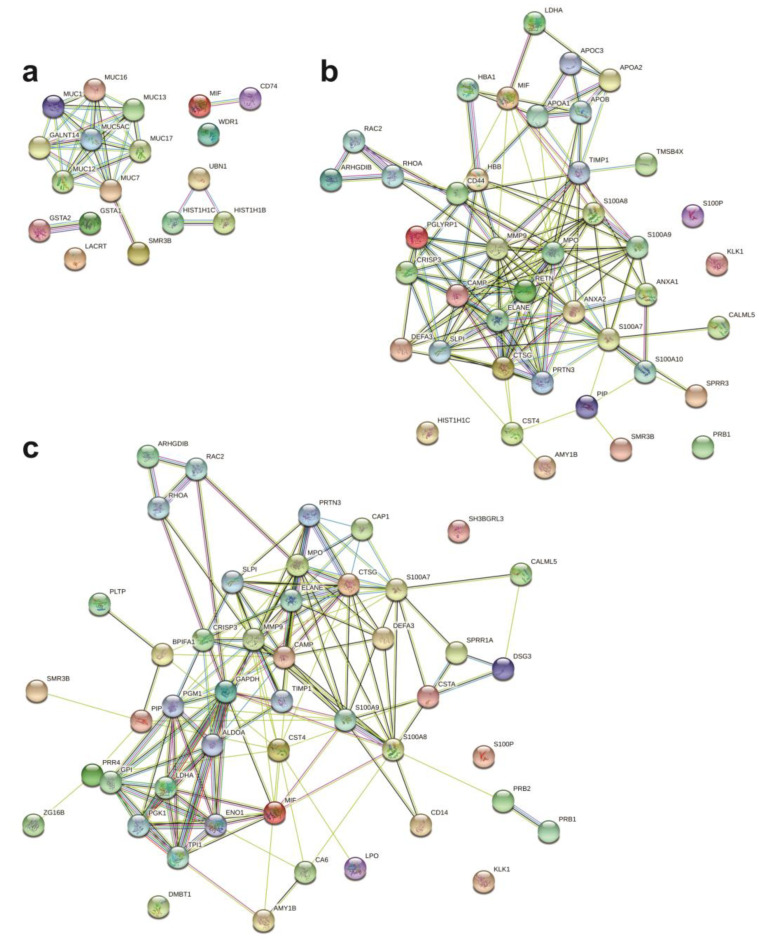
Protein–protein interaction network of significantly altered proteins in induced sputum by STRING 11.0. Eosinophilic (**a**), neutrophilic (**b**), and mixed granulocytic (**c**) asthma. The proteins are represented by the nodes, and the lines represent the interactions with predicted interactors.

The protein–protein interaction analysis was performed with the differentially expressed serum and sputum proteins separated by the phenotypic group in which the proteins were upregulated. Four disconnected clusters were formed when analyzing the eosinophilic altered sputum proteins (Figure 4a) involved in several pathways, such as the innate immune system, hemostasis, and activation of DNA fragmentation factor (Supplementary Figure S1a). Only one large cluster was formed between altered sputum proteins from the neutrophilic group, as well as in the mixed granulocytic group (Figure 4b,c). In both clusters, proteins were mostly involved in the innate immune system, antimicrobial peptides, and neutrophil degranulation (Supplementary Figure S1a).

**Figure 5:**
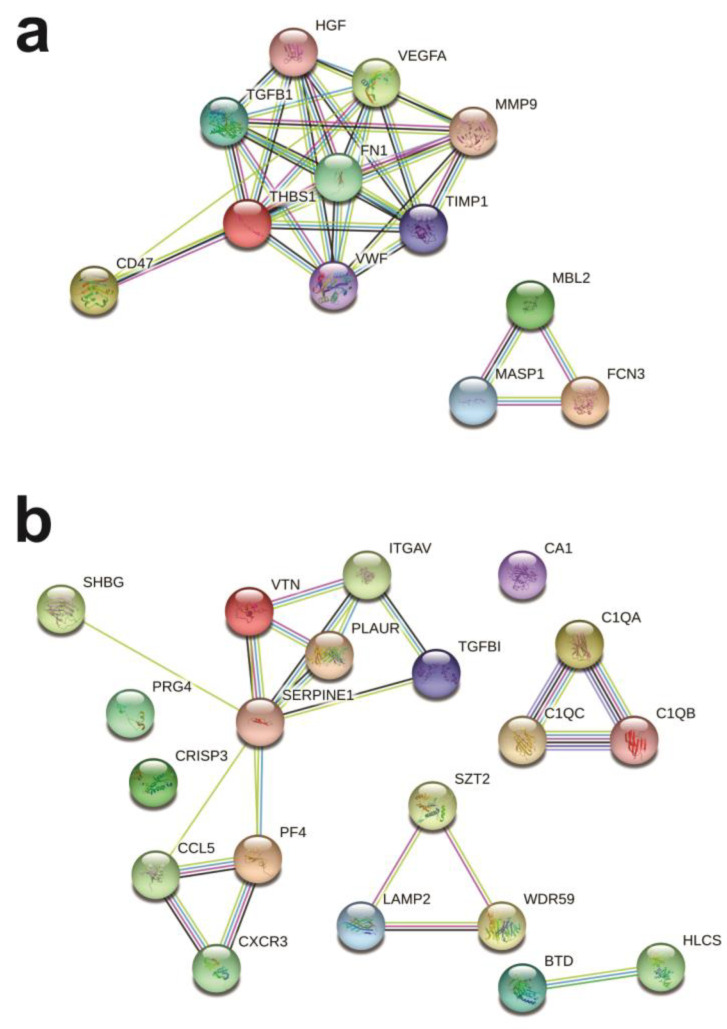
Protein–protein interaction network of significantly altered proteins in serum by STRING 11.0. Eosinophilic (**a**) and mixed granulocytic (**b**) asthma. The proteins are represented by the nodes, and the lines represent the interactions with predicted interactors.

The network formed by upregulated serum proteins from eosinophilic patients showed two disconnected clusters (Figure 5a) associated with the immune system, the lectin pathway of complement activation, and platelet degranulation (Supplementary Figure S1b). By contrast, two clusters were tightly connected, and three independent clusters were formed in the network of altered serum proteins from the mixed granulocytic group (Figure 5b). Interestingly, these proteins were involved in similar pathways found in mixed granulocytic sputum, as in the innate immune system and neutrophil degranulation but also others like homeostasis and platelet degranulation (Supplementary Figure S1b).

## 3. Discussion

Asthma phenotyping based on the airway inflammatory profile is crucial for personalized treatment definition since, in most cases, patients from different phenotypes do not respond satisfactorily to the same therapy. Moreover, classifying different clinical and inflammatory forms could result in the identification of new therapeutic targets. Proteomic analysis is an important technique for identifying biomarkers. These molecules may be useful in the diagnosis of these patients, as well as assist in the treatment and monitoring of the disease. In this study, proteomic analysis was employed to classify eosinophilic, neutrophilic, mixed granulocytic, and paucigranulocytic asthma inflammatory patterns.

We identified a similar protein expression pattern between neutrophilic and mixed granulocytic induced sputum, which differs significantly from the patterns of the other groups. Contrastingly, minor differences were found in the protein expression pattern from serum proteins. In this way, it was possible to develop a potential biomarker panel to assist the diagnosis of the asthma phenotypes using induced sputum proteins. The protein–protein interactome evidenced that many of these proteins, especially from neutrophilic and mixed granulocytic groups, are functionally linked to each other. 

Similar clinical and inflammatory characteristics between asthma groups were detected with no differences in any analyzed parameter, including atopy, age at onset, and FEV1 (% Predicted). Interestingly, sputum cell count did not reflect blood cell count, as no difference was found in blood eosinophils and neutrophils. Blood eosinophil count has been proposed as a biomarker for airway eosinophilia due to correlations between sputum and blood cell counts [21,22,23]. However, as eosinophil levels are not constant, only one measurement can lead to an incorrect diagnosis, especially if patients are taking systemic corticosteroids [14,21,22,24]. 

Among the proteins analyzed in our study, a small amount was present in both blood and induced sputum. Despite this large difference between the airway proteins and those found systemically, their molecular functions and biologic processes remained the same in the two environments. Curiously, prior research identified that proteins from sputum proteome and bronchoalveolar lavage fluid of asthmatic patients had similar genetic ontology. Also, they found the same molecular functions and biologic processes we demonstrated [25,26].

Among the promising proteins selected in induced sputum, CAMP and ANXA1 were found to be upregulated in the neutrophilic group. CAMP is released by neutrophils during inflammation to host defense [27], although its role in asthma has been little investigated [28,29,30]. This protein was found to be downregulated in asthmatic sputum patients compared to the control group in a prior comparative study among asthma, COPD, and cystic fibrosis patients. However, the sputum inflammatory cellular profile was investigated only in cystic fibrosis that presented higher levels of CAMP—consequently, with a high count of neutrophils [30]. ANXA1 is an anti-inflammatory mediator that reduces neutrophil migration [31] and could be acting to regulate the inflammation generated by the high number of neutrophils in neutrophilic asthma. Many studies have demonstrated its important role in asthma, and even suggested ANXA1 as a therapeutic target for this pathology [32,33,34]. Nevertheless, just recently, it was shown to be a predictive protein for neutrophilic asthma, although it was not different between the other asthma phenotypes [13].

Discussions regarding the role of BPIFA1 in asthma have increased in recent years, evaluating its immune modulator function and its potential as a novel therapy [35,36,37,38,39]. BPIFA1 is secreted by the airway epithelial cells controlling the contraction of the airway smooth muscle and, therefore, hyperresponsiveness. Interestingly, cytokines related to Th2 immune response can inhibit this protein, promoting increased contraction and hyperresponsiveness [36], which corroborates with its downregulated levels observed in the eosinophilic group in our study. By contrast, we demonstrated that MUC5AC was upregulated in eosinophilic asthma. MUC5AC was previously correlated with asthma, since it is induced by the Th2 immune response triggered by allergic eosinophilic asthma [40,41,42]. 

Although it was not possible to develop a biomarker panel from serum proteins that could distinguish all the four asthma phenotypes, important proteins could significantly distinguish some phenotypes, as FCN3 was detected in different levels between eosinophilic and neutrophilic asthma. This protein is implicated in the activation of complement via the lectin pathway enhancing phagocytosis [43], and its deficiency may be associated with increased infection. High levels of this protein were detected in the serum of refractory asthma patients in relation to non-refractory asthma [44], but it has not yet been associated with asthma phenotypes. The THBS1 gene has been implicated in the response to stress in asthma [45,46] and in the cellular immunity activity in allergic asthma [47], corroborating with the high levels in eosinophilic asthma demonstrated in our study. 

CRISP3 is present in neutrophils granules and possibly plays an antimicrobial role [48,49]. Despite this, this protein was not increased in the serum of the neutrophilic group but was found to be upregulated in the serum of mixed granulocytic group compared with the eosinophilic and neutrophilic groups. However, it is important to note that none of the groups showed a difference in blood neutrophil count. Although there is a lack of information about this protein and few investigations of asthma, previous studies evidenced high gene expression in severe asthmatics compared with non-asthmatic individuals [50] and in atopic asthmatics compared with healthy non-atopic individuals [51]. According to our data, LAMP2 could segregate mixed granulocytic from paucigranulocytic asthma. This protein acts on the degranulation of neutrophils and platelets, present in neutrophil granules [52], and on homeostasis, especially autophagy [53]. There is also little information about the role of LAMP2 in asthma; however, it was recently linked with this disease but not with the phenotypes [54,55]. 

## 4. Materials and Methods

### 4.1. Subject Recruitment

Thirty-two asthmatic adult subjects diagnosed as defined by the Global Initiative for Asthma (GINA) [1] guideline were enrolled in this study, comprising eight patients per each group (eosinophilic asthma, neutrophilic asthma, paucigranulocytic asthma, and mixed granulocytic asthma). Participants were excluded if they were current smokers or had a history of more than 10 pack years, had already used immunosuppressants or immunomodulators, had a severe exacerbation and respiratory tract infection within 4 weeks of sample collection, or any clinically important comorbidity. During the visit, induced sputum and blood samples were collected on the same day, and clinical measurements were recorded. The Ethics Committee of the Federal University of São Paulo approved the study protocols, and written informed consent was obtained from all participants (N. 0932/15). All methods were performed in accordance with the relevant guidelines and regulations.

### 4.2. Sample Collection and Phenotyping

Sputum was induced according to the method of Pizzichini [56,57]. Sputum was processed immediately; the cell-free supernatant was stored at −80 °C until proteomic analysis, and cell cytospins were prepared for the differential count. The sputum eosinophil counts were set at 3% and greater and neutrophil counts at 65% and greater as a cut-off point to define eosinophilic and neutrophilic phenotypes, respectively. The mixed granulocytic phenotype was defined by both eosinophil and neutrophil count in the cut-off point or higher, whereas the paucigranulocytic group was identified by less than 3% eosinophils and 65% neutrophils. The blood sample was also collected from all patients, centrifuged to obtain the serum, aliquoted, and stored at −80 °C until proteomic analysis. 

### 4.3. Sample Preparation

Sera were immunodepleted for 14 highly abundant serum proteins using Multiple Affinity Removal Column-Human 14 (Agilent Technologies, Santa Clara, CA, USA) on an Akta Purifier HPLC (GE Amersham, Amersham, UK) according to the manufacturer’s protocol. Serum proteins (1 µg) and induced sputum proteins (1 µg) were reduced, alkylated, and digested with ProteoExtract All-in-One Trypsin Digestion Kit (EMD Millipore, Billerica, MA, USA) and desalted with ZipTip C18 (Pierce, Thermo Fisher Scientific, Bremen, Germany).

### 4.4. Proteomic Analysis

Tryptic peptides were separated by reverse-phase nano-HPLC (Dionex Ultimate 3000, Thermo Fisher Scientific, Bremen, Germany; column: Acclaim PepMap RSLC C18, 75 µm × 15 cm, Dionex, Thermo Fisher Scientific, Bremen, Germany). The column was eluted with an acetonitrile gradient (Solvent A: 0.1% (*v*/*v*) FA/0.01% (*v*/*v*) TFA/5% (*v*/*v*) DMSO; solvent B: 0.1% (*v*/*v*) FA/0.01% (*v*/*v*) TFA/90% (*v*/*v*) ACN/5% (*v*/*v*) DMSO; 5–45% B in 60 min) at a flow rate of 0.3 µL/min at 55 °C. Peptides were analyzed with a Q Exactive Orbitrap mass spectrometer (Thermo Fisher Scientific, Bremen, Germany) directly coupled to the HPLC. The capillary voltage at the nano electrospray head was 2 kV; the instrument was tuned for maximum sensitivity. For peptide assignments, a top 12 method was used with normalized fragmentation energy at 27%. Protein assignment was conducted with PEAKS Studio X (Bioinformatics Solutions, Waterloo, ON, Canada). Spectra were searched against the Uniprot database (Homo sapiens, January 2019 version). Only peptide hits with a probability score (−10 logP) ≥ 20 and a false discovery rate (FDR) of 1% were used for protein identification. Label-free quantification was evaluated by PEAKS Q of PEAKS Studio X, using the area under the curve (AUC) of chromatographic peaks from each sample. Data were normalized and compared for relative abundance.

### 4.5. Functional and Network Analysis

Gene ontology analysis of induced sputum and serum proteins was obtained from the database Panther 14.1: http://www.pantherdb.org/ (accessed on 4 April 2023). The molecular function and biological process from the identified proteins were compared between the phenotypic groups.

The protein network was constructed to verify protein–protein interaction using database STRING 11.0: http://string-db.org/ (accessed on 16 May 2023). Differentially expressed proteins were analyzed in this web tool, with a medium confidence score of >0.4, allowing up to 10 interactors. 

### 4.6. Statistical Analysis

Statistical analysis of clinical data was assessed using Kruskall Wallis and Dunn’s post hoc test. Protein differences between the groups were analyzed using multiple *t*-tests between every two groups. All the data obtained were analyzed using Prism 8.0 (GraphPad Software, Inc., San Diego, CA, USA). Volcano plots and heatmaps were also generated using the same software. *p*-values less than 0.05 were considered significant.

## 5. Conclusions

This study performed a comparative proteomic analysis in both induced sputum and serum from eosinophilic, neutrophilic, paucigranulocytic, and mixed granulocytic asthma patients for the first time. Our study demonstrates that induced sputum and serum profiling can describe complex inflammatory asthma phenotypes. We identified a potential five-biomarker panel (BPIFA1, MUC5AC, CAMP, CTSG, and ANXA1) that illustrates the inflammatory activity of asthma in sputum and could improve asthma phenotyping, providing valuable insights into personalized treatment approaches. Although it was not possible to find proteins that could discriminate all the asthma phenotypes in serum, FCN3 is notable, as it could discriminate eosinophilic from the neutrophilic and mixed granulocytic groups. Despite the fact that many of these proteins have already been associated with asthma, a few of them led to inflammatory phenotypes. The specific roles of certain proteins on asthma pathogenesis have only recently begun to be investigated and should be further investigated, given their potential use as targets for therapy and their potential to enhance the understanding of asthma pathophysiology. Notably, the limitations of this study include the lack of validation of these proteins using alternative methods and a relatively small sample size, demanding further investigation with larger cohorts and validation through diverse immunological approaches to solidify our findings.

## Figures and Tables

**Table 1:** Characteristics of study subjects.

	E	N	M	P	*p* Value
	(*n* = 8)	(*n* = 8)	(*n* = 8)	(*n* = 8)	
Age, years	48.6 ± 21.2	56.1 ± 13.7	55.5 ±10.0	48.9 ± 14.8	0.43
Female sex, n (%)	4 (50)	4 (50)	6 (75)	5 (62.5)	0.71
BMI, kg/m^2^	25.1 ± 5.9	25.9 ± 2.3	28.9 ± 5.2	26.2 ± 5.3	0.57
Age at onset, ≥12 years	5 (62.5)	3 (37.5)	6 (75)	5 (62.5)	0.50
SPT positivity, n (%)	6 (75)	8 (100)	5 (62.5)	5 (62.5)	0.27
FEV_1_, % predicted	73.9 ± 12.5	71.5 ± 21.4	74.3 ± 15.6	78.5 ± 10.9	0.93
Sputum eosinophils, %	25 (12.5–36.7)	0.5 (0–1.2)	10 (4.9–13.2)	0.5 (0–1)	<0.0001 ^a,b,c^
Sputum neutrophils, %	47 (40.5–60.2)	94.5 (89.7–95.7)	80.5 (76–84.5)	43 (20.2–60.7)	<0.0001 ^a,c,d^
Blood eosinophils/µL	303.5 (171.2–511.2)	144 (102.7–246.5)	137.5 (115–207.7)	286.5 (102.2–450)	0.25
Blood neutrophils/µL	4232 (2948.7–7220.7)	4250 (3827.5–4596.5)	3831.5 (3303.5–4541.5)	3612 (2513.5–4513.7)	0.79
GINA Step					
3, n (%)	0 (0)	0 (0)	0 (0)	1 (12.5)	0.53
4, n (%)	4 (50)	4 (50)	4 (50)	5 (62.5)	
5, n (%)	4 (50)	4 (50)	4 (50)	2 (25)	
Inhaled corticosteroid, mcg	1000 (800–1200)	1000 (800–1200)	1200 (950–1200)	800 (700–900)	0.79

Data are presented as means ± SDs, n (%) or as medians (interquartile ranges), unless otherwise specified. E, eosinophilic asthma; N, neutrophilic asthma; P, paucigranulocytic asthma; M, mixed granulocytic asthma; BMI, body mass index; SPT = skin prick test; FEV**_1_**, forced expiratory volume in 1 s. ^a^ E vs. N, ^b^ E vs. P, ^c^ M vs. P, ^d^ N vs. P.

## Data Availability

Data are contained within the article and Supplementary Materials.

## Supplementary Materials

The supporting information can be downloaded at https://www.mdpi.com/article/10.3390/ijms25063501/s1.
