# Supplementary material for: Exploring Inflammatory Asthma Phenotypes: Proteomic Signatures in Serum and Induced Sputum
Source: Int J Mol Sci. 2024 Mar 20;25(6):3501. doi: 10.3390/ijms25063501 (PMC10971300; doi:10.3390/ijms25063501)
Supplement: Supplementary file 1 [file ijms-25-03501-s001.zip › ijms-2874838-supplementary.pdf]

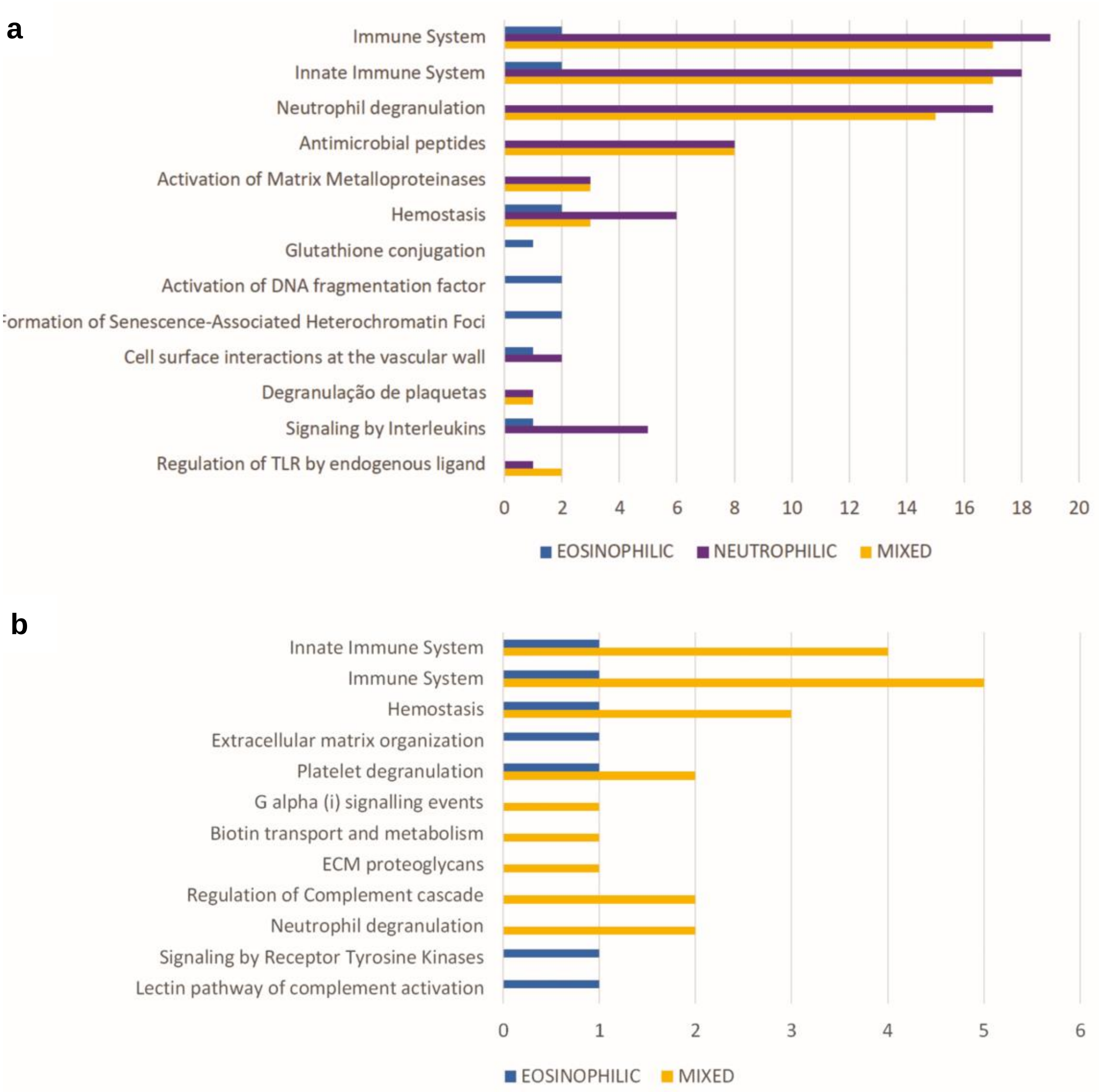

Supplementary Figure S1. Main enriched pathways of significantly altered proteins in induced sputum **(a)** and serum **(b)** of patients with eosinophilic (blue), neutrophilic (purple) and mixed (yellow) asthma. The number of significantly different proteins associated with each pathway identified by the database Reactome is shown in the bar graph.

Supplementary Table S1. List of the significantly different proteins in induced sputum of patients with eosinophilic, neutrophilic, mixed and paucigranulocytic asthma.

**Gene symbol**	**Description**	**Log Dif (E vs N)**	**Log Dif (E vs M)**	**Log Dif (E vs P)**	**Log Dif (N vs M)**	**Log Dif (N vs P)**	**Log Dif (M vs P)**
S100P	Protein S100-P	-4,18***	-3,42**	NS	NS	NS	NS
S100A8	Protein S100-A8	-2,96*	-1,87*	NS	NS	4,38***	3,28***
CAMP	Cathelicidin antimicrobial peptide	-13,08*	NS	NS	NS	17,32**	13,25*
PGLYRP1	Peptidoglycan recognition protein 1	-12,46*	NS	NS	NS	14,92**	NS
PIP	Prolactin-inducible protein	-1,64*	-2,81***	NS	NS	NS	NS
AMYA1	Alpha-amylase 1	-4,046*	-5,55**	NS	NS	NS	2,93*
CST4	Cystatin-S	-2,10*	-2,76*	NS	NS	2,58**	3,23**
KLK1	Kallikrein-1	-2,78*	-3,11**	NS	NS	NS	1,58*
TMSB4X	Thymosin beta-4	-2,44*	NS	NS	NS	NS	NS
CRISP3	Cysteine-rich secretory protein 3	-1,67*	-1,80*	NS	NS	2,616*	2,74*
GSTA1	Glutathione S-transferase A1	11,84*	NS	NS	NS	NS	NS
DSG3	Desmoglein-3	NS	-13,35**	NS	NS	NS	14,43**
PRRL4	Proline-rich protein 4	NS	-2,77*	NS	NS	NS	NS
LPO	Lactoperoxidase	NS	-1,29*	NS	NS	NS	NS
BPIFA1	BPI fold-containing family A member 1	NS	-10,70*	NS	-2,95*	NS	NS
ZG16B	Zymogen granule protein 16 homolog B	NS	-2,04*	NS	NS	NS	NS
CA6	Carbonic anhydrase 6	NS	-2,99*	NS	NS	NS	NS
MIF	Macrophage migration inhibitory factor	NS	12,84*	NS	13,86*	NS	NS
MUC5AC	Mucin-5AC	NS	NS	13,41*	NS	NS	NS
SMR3B	Submaxillary gland androgen-regulated protein 3B	NS	NS	3,23*	NS	2,82*	4,13**
H1-5	Histone H1.5	NS	NS	13,12*	NS	NS	NS
LACRT	Extracellular glycoprotein lacritin	NS	NS	13,34*	NS	NS	NS
BASP1	Brain acid soluble protein 1	NS	NS	11,47*	NS	NS	NS
H1-2	Histone H1.2	NS	NS	10,68*	NS	11,33*	NS
IGHV3-66	Immunoglobulin heavy variable 3-66	NS	NS	NS	-2,64*	NS	NS
ANXA1	Annexin A1	NS	NS	NS	2,47*	9,21*	NS
ELANE	Neutrophil elastase	NS	NS	NS	NS	4,23**	3,01**
PRB1	Basic salivary proline-rich protein 1	NS	NS	NS	NS	20,09**	NS
APOA2	Apolipoprotein A-II	NS	NS	NS	NS	15,08**	NS
CTSG	Cathepsin G	NS	NS	NS	NS	15,33**	13,77*
CALML5	Calmodulin-like protein	NS	NS	NS	NS	13,58**	13,48**

	5						
DEFA3	Neutrophil defensin 3	NS	NS	NS	NS	3,44**	2,90**
MPO	Myeloperoxidase	NS	NS	NS	NS	3,52*	2,88**
LDHA	L-lactate dehydrogenase A	NS	NS	NS	NS	12,41*	11,97*
PRTN3	Myeloblastin	NS	NS	NS	NS	3,26*	2,80**
HBB	Hemoglobin subunit beta	NS	NS	NS	NS	12,61*	NS
ANXA2	Annexin A2	NS	NS	NS	NS	10,96*	NS
RETN	Resistin	NS	NS	NS	NS	11,17*	NS
S100A7	Protein S100-A7	NS	NS	NS	NS	10,73*	9,99*
CD44	CD44 antigen	NS	NS	NS	NS	12,33*	NS
ARHGDIB	Rho GDP-dissociation inhibitor 2	NS	NS	NS	NS	9,70*	9,04*
SPRR3	Small proline-rich protein 3	NS	NS	NS	NS	9,44*	NS
MMP9	Matrix metalloproteinase-9	NS	NS	NS	NS	7,41*	7,04*
DMBT1	Deleted in malignant brain tumors 1 protein	NS	NS	NS	NS	NS	1,32*
CD14	Monocyte differentiation antigen CD14	NS	NS	NS	NS	NS	10,93*
GAPDH	Glyceraldehyde-3-phosphate dehydrogenase	NS	NS	NS	NS	NS	2,55*
SH3BGRL3	SH3 domain-binding glutamic acid-rich-like protein 3	NS	NS	NS	NS	NS	10,85*
PRB2	Basic salivary proline-rich protein 2	NS	NS	NS	NS	NS	13,17*
GPI	Glucose-6-phosphate isomerase	NS	NS	NS	NS	NS	11,04*
HV64D	Immunoglobulin heavy variable 3-64D	NS	NS	NS	NS	NS	10,74*
CAP1	Adenylyl cyclase-associated protein 1	NS	NS	NS	NS	NS	9,02*
CSTA	Cystatin-A	NS	NS	NS	NS	NS	11,31*

* $p < 0,05$. ** $p < 0,01$. *** $p < 0,001$.
E, eosinophilic asthma; N, neutrophilic asthma; P, paucigranulocytic asthma; M, mixed granulocytic asthma; NS, not significant.

Supplementary Table S2. List of the significantly different proteins in serum of patients with eosinophilic, neutrophilic, mixed and paucigranulocytic asthma.

Gene symbol	Description	Log Dif (E vs N)	Log Dif (E vs M)	Log Dif (E vs P)	Log Dif (N vs M)	Log Dif (N vs P)	Log Dif (M vs P)
FCN3	Ficolin-3	12,83*	9,55*	NS	NS	NS	NS
CA1	Carbonic anhydrase 1	NS	-3,20*	NS	NS	NS	NS
C1QA	Complement C1q subcomponent subunit A	NS	NS	NS	NS	NS	2,51*
PF4	Platelet factor 4	NS	-3,86**	NS	-3,22**	NS	NS
VTN	Vitronectin	NS	-1,15*	NS	-1,80**	NS	NS
SHBG	Sex hormone-binding globulin	NS	-2,88*	NS	NS	NS	NS
THBS1	Thrombospondin-1	NS	NS	10,92*	NS	NS	NS
LAMP2	Lysosome-associated membrane glycoprotein 2	NS	NS	NS	NS	NS	10,70*
BTD	Biotinidase	NS	NS	NS	NS	NS	2,00*
CRISP3	Cysteine-rich secretory protein 3	NS	-9,93*	NS	-8,58*	NS	NS
TGFBI	Transforming growth factor-beta-induced protein	NS	NS	NS	NS	NS	10,37*
PRG4	Proteoglycan 4	NS	NS	NS	NS	NS	8,32*

* p < 0,05. ** p < 0,01.

E, eosinophilic asthma; N, neutrophilic asthma; P, paucigranulocytic asthma; M, mixed granulocytic asthma; NS, not significant.
